# 

**Published:** 1907-01-15

**Authors:** 


### SUBJECT INDEX TO VOLUME LXI.

EVENT AND COMMENT.

Banquet to George L. Field, 539.

Black, Professor G. V., Banquetted, 57.

Commission Fees, 58.

Definite Dosage, 163.

Dental Anesthetics, 217.

Dental Text Books, 3.

Detroit Dental Societies' Annual Clinic, 109.

Evils of Tooth Brushes and Powders, 55.

Independent Dental Journalism, 431

Institute of Dental Pedagogics, 1.

Michigan's New Dental Law, 325.

Michigan State Dental Society, 323.

Miller, Death of Professor W. D., 377.

Occlusion in Crown and Bridge Work 164.

Officers of Michigan State Dental Society, 324.

Oral Hygiene for School Children, 485.

Prohibition, State Board Examinations, 4.

Smith, Dr. H. A., Fifty Years in Practice, 269.

Taggart's Cast Inlay, 110.

Tooth Roots, 593.

ORIGINAL PAPERS.

Actinomycosis, George Zederbaum, 460.

Address, H. L. Ambler, President Ohio State Dental Society, 283.

Amalgams, their Abuses and Treatment, N. K. Garhart, 285.

Bandless Richmond Crown, J. C. Longfellow, 308.

Base for Richmond Crown, James E. Boyd, 313.

Combination Gold Faced Platinum Crown, with Porcelain Cusp and Face, S. A. Stuart, 310.

Crown with Invisible Band, S. D. Ruggles, 306.

Dental Anesthetics, S. Straith, 112.

Dental Army Service in the Philippines, S. D. Boak, 289.

Detrimental Physical Properties of Expansion and Compression of Plaster of Paris, J. H. Prothero, 22.

Discourse on Brains, C. M. Wright, 384.

Facial Expression, Mechanical Methods of Correcting, W. A. Price, 300

Flexible Rubber Retention, N. H. Grove, 307.

Gold Inlays, C. H. Worboys, 59.

Gold Inlays, T. P. Hinman, 301.

Gold Inlay, H. Barnes, 312.

Incident in Porcelain Work, J. M. Thompson, 177.

Method of Retaining Teeth after Regulating, W. S. Locke, 276.

Mistakes and Failures in Bridgework and Some Remedies, F. E. Logan, 223.

Modern Dental Remedies, E. T. Loeffler, 68.

Oral Prophylaxis, W. H. VanDeman 296.

Porcelain Jacket Crowns, J. E. Roache, 454.

Porcelain, Practical Points About, E. H. Shannon, 298.

Porcelain, Some Practical Suggestions, E. C. Sherman, 299.

Pressure Anesthesia, Charles G. Hampton, 568.

Propogandism of Dental Education, in Our Public Schools, Geo. Zederbaum, 487.

Pulp, and Its Pathology, R. W Bunting, 468.

Putrescent Teeth, Treatment of, L. P. Hall, 329

Remoldable Porcelain, C. H. Land, 595.

Reorganization of Illinois State Dental Society, A. D. Black, 5.

Slipjoint Attachment to Hand-piece for Wetting Stones, D. Stern, 311.

Treatment of Pyorrhoea Alveolaris G. P. Rogers, 434.

Why the Profession should Adopt Porcelain as its Standard Filling Material, W. T. Reeves, 166.

REPRINTS.

Alcohol in Mouth Disinfection, W. F. Litch, 404.

Artificial Enamel, R. B. Tuller, 512

Brightening Orthodontic Appliances, W. J. Brady. 613.

Business Methods, J. H. Irvin, 605.

Cast Inlays, W. H. Taggart, 600.

Cast Inlays and Bridges, 364.

Cocaine, New Way to Apply, F. Touchard, 139.

Compressed Air, W. F. Linnert, 417.

Dentine, with Special Reference to the Production of Anesthesia, by Pressure, W. D. Miller, 187.

Electro Gilding, J. T. Barker, 474.

Evolution of the Inlay, F. W. McDonald, 456.

Extracting Abscessed Teeth, G. B. Mitchell, 390.

Gold Inlays, C. F. Woodbury, 408.

Gold Inlay, D. D. Campbell, 525.

Inlay Matrix, J. Q. Byram, 522.

Liquid Rubber, J. T. Nelson, 414.

Management of a Dental Practice E. C. Briggs, 351.

Pressure Anesthesia, H. Prinz, 250.

Porcelain Misconceptions, L. Ladewich, 519.

Sanitation of Cement for Filling Crowns and Bridges, W. J. Emelin, 472.

The Schools, and Children's Teeth, C. O. Kimball, 500.

WITH OUR EDITORS.

A Duty to the Profession, C. N. Johnson, 86.

Anesthetics in General, and Cocaine in Particular, E. C. Kirk, 39.

Attainment of Asepsis in Dental Practice, 146.

Banquet to G. V. Black, 159.

Better Society Organization, J. N. Crouse, 199.

Case vs. Dewey, Angle, et. al., W. J. Brady, 362.

Dental Labor Union Threatened, R. Ottolengui, 82.

Drawing the Line, F. G. Wortley, 197.

Extracting for Regulating, W. J. Brady, 194.

Fads, F. O. Hetrick, 360.

Faulty Education, Geo. W. Cook, 87.

Limitation of Dentistry to its Mechanics, G. H. Wilson, 37.

Some things Lacking in Dental Prosthesis, S. H. Guilford, 76.

System, F. O. Hetrick, 79.

The Opportunities of Today, J. N. Crouse, 86.

Your Vacation, T. J. Collins, 200.

OBITUARY.

Uriah D. Billmeyer, 54.

Mrs. C. F. W. Bodecker, 374.

Theodore D. Buhl, 255.

Professor W. D. Miller, 377.

Wm. N. Fuller, 315.

BIBLIOGRAPHY.

American Text Book of Prosthetic Dentistry, by Charles R. Turner 202.

Anatomical Nomenclature, by L. F. Barker, 201.

Atlas and Text Book of Dentistry, by Gustav Preiswork, 89.

Dental Formulary, by Herman Prinz, 478.

Proceedings National Dental Society 479.

ANNOUNCEMENTS.

American Dental Society of Europe, 108, 321.

American Medical Association, 268.

American Society of Orthodontists, 266.

Arkansas State Board of Examiners, 216.

California State Board of Examiners 161.

Chicago Odontographic Society, Officers, 162.

Dennis, Dr., Married, 108.

Detroit Dental College Clinic, 216.

Essayists for Jamestown Convention 268.

Florida State Board of Examiners, 265.

Garhart Dental Manufacturing Co., 105.

Gorman, Dr. J. A., Removal, 162.

Illinois State Dental Association, 427.

Indiana State Dental Association, 216, 267, 374.

Institute of Dental Pedagogies, 592, 637.

Jamestown Dental Convention, 105, 259, 374, 428.

Kentucky State Dental Association, 105, 162, 216, 427.

Kentucky State Board of Examiners, 267.

Lake Erie Dental Association, 215,

Lebanon Valley Dental Association, 426.

Lodge, Dr. E. B., Married, 107.

Michigan State Dental Association, 215, 264, 266, 324.

Michigan State Board of Dental Examiners, 267, 326, 537.

Missouri State Dental Association, 215, 268.

National Dental Society, 52, 268, 319, 428, 538.

National Association of Dental Faculties, 268, 538.

National Association of Dental Examiners, 161, 267, 537.

Nebraska State Board of Dental Examiners, 216.

New Editor "Dental Digest," 636

New Jersey Dental Examinors, 592.

New York State Dental Society, 162,

New York Seventh District Society 163.

Ohio Board of Examiners, 591.

Ohio State Board of Dental Examiners, 266, 320.

Ohio State Dental Society, 590.

Ohio State Dental Society, 591.

Resolution on Death of Prof. Miller, 591.

San Francisco Relief Committee, 320.

Southern California Dental Association, 108.

Southwestern Michigan Dental Society, 265.

Saint Louis Dental Societies, Consolidated, 104.

St. Louis Dental Society, 637.

Tennessee State Dental Association, 216.

West Virginia State Board of Examiners, 267.

West Virginia State Dental Association, 427.

West Virginia State Dental Society, 637.

Wyoming State Board of Examiners, 319.

Virginia State Dental Association' 318.

INDENTS.

Adrenalin, Chloride, Objection, 209.

Amalgam Repairs, 256.

Anesthetizing Inflamed Pulps, 626.

An Extraordinary "Mechanical Face," 632.

Are We Now a Profession, 208.

Artifi ial Dentures Shovld Duplicate Nature, 635.

Baby has Teeth at Birth: may become Rival of Roosevelt, 618.

Bicuspids and Molars, 532

Bite, Correct One, 369.

Beveled Walls, 625.

Codrenin, 634.

Conservation vs. Practicability, 205.

Contour Without Exaggeration 424.

Copper Matrices, 92.

Cut Solder Large, 205.

Cuttirfg Out Fissures, 622.

Decay of Children's Teeth, 617.

Decent Cleanliness, 94.

Dental Clinic in Schoo, 625.

Deodorizer for Office, 368.

Defective Enamel, 204.

Dentist Restrained, 623.

Denture Adhesion, 211.

Deodorizers, 157.

Die Metal for Modeling Compound Impressions, 205.

Diseased Molar Sockets, 480.

Don't Promise, 627.

Echinacea, 621.

Editors of Trade Dental Journals, 372.

Efficiency, 47.

Electric Sleep, 421.

Ethics, 45.

Ethyl Chloride Fatalities, 93.

Evils Incidedt to Use of tqe Dam, 628.

Evolution of the Student, 45.

Extracting Live Pulps, 209.

Extracting for Regulating, 630.

Extracting Abscessed Teeth, 258.

Facial Maldevelopement, 97.

Fatalities, 257.

Files, Cleaning, 257.

Formalin, 370.

Fragrant Mouth Wash, 157.

Full Impression with Compound and Plaster, 317.

Fusible Alloy Tooth Form, 371.

Gold Inlay, Original Method, 50.

Gold Inlay, 94, 100, 208, 530.

Handpiece, Care of, 256.

Higher Ideals, 423.

Hot Brick, 257.

How Often Should Orthodontia Patients be Seen, 623.

How the Ancients Ate, 91.

How to Obtain a Correct Bite, 624

Immediate or Delayed Plate Insertion, 425.

Inspection of School Children's Teeth, 629.

Instantaneous Relief in Cases of Blind Abscess, 629.

Investment Plaster, 369.

Light for the Dentists, 102.

Local Anesthetic for Pyorrhea, 619.

Lower Impressions, 316.

Lower Partial Plates, 368.

Lower Denture Retention, 157.

Low-Fusing Alloy, 617.

Method of Setting an Inlay, 206.

Making Cements, 627.

Neck Broken by Dentist, 633.

Neckwear for Dentists, 46.

Non-Corosive Sterilizer for Dental Instruments, 631.

Nose Blowing, 480.

Obtunding Sensitive Dentine, 204.

Obtunding Pain in Children's teeth, 420.

Painless Methods, 49.

Petrogen, 206.

Phenol and Camphor in Suppurations, 95.

Preserve the Root, 368.

Pressure Anesthesia and Cataphorsis Identical, 256.

Pressure Anesthesia, 624.

Porcelain Baking, 528.

Putrescence in Pulp Chamber or Root Canal, 618.

Putrid Canals, 96.

Pyorrhea Pockets, 619.

Pyorrhoea Treatment, 209.

Pyorrhoea by Opsonins, 527.

Repairing Gold Fillings, 368.

Replacing Facings, 93.

Removing Broken Pivots, 207.

Separating Gold, etc., 483.

Seamless Crowns, 529.

Sensitive Dentin, 92, 206, 620.

Setting Inlays, 622.

Silk First, Then Clamps, 204.

Silver Nitrate in Pyorrhea, 619.

Silver Nitrate Stains, 157.

Soldering Aluminum, 617.

Somnoform, 535.

Stanno-Percha, 204.

Sterilizing Impression Trays and Impressions, 205.

Sterilizing of Dentine, 257.

Sticky Wax Moulding, 422.

Sulpho Carbolate of Zinc, 481.

Taggart Cast Inlay, 158.

Taking Impressions, 619.

Testing and Electric Furnaces, 91.

The Clasp, 617.

The Lower Plate, 533.

The Noble Prizes, 99.

Therapeutical Notes, 420.

The Retention of Upper Dentures, 629.

Third Molar Extraction, 212.

To Make a Hard Model, 617.

To Obtund Sensitive Dentin, 618.

To Re-bake an Inlay, 621.

Tropa-Cocain, 623.

Troubles of Various Dentists, 209.

Treating Putrescence in Roots, 207

Treatment of Sensitive Palates, 207.

Treatment of Sensitive Palates prior to the Taking of Plaster Impressions, 620.

Upper Impressions, 256.

Vulcanizing Rubber, 620.

Wax, to Clean, 257.

AUTOGRAPHIC.

Allen, C. C., 205.

Ames, W. V.-B., 368.

Ambler, H. L., 283.

Andrews, R. R., 204.

Badcock, J. H., 205.

Barker, G. T., 96.

Barker, J. T., 474.

Barker, L. F., 201.

Barnes, V., 281.

Barnes, H., 51, 312, 305.

Beavers, E. A., 133.

Billmeyer, W. D., 54.

Bitting, Rev. W. C., 153.

Black, G. V., 398, 152.

Black, A. D., 5, 16.

Blackmarr, C. B., 13.

Bogue, E. A., 92.

Boak, D. D., 289.

Bowman, G. A., 152.

Boyd, J. E., 313.

Bowles, G. C., 67.

Brady, W. J., 194, 364, 623.

Brashear, K. C., 126.

Brideford, J. L., 533.

Briggs, C. P., 206, 207.

Briggs, E. C., 351.

Brigham, O. F., 257.

Brown, G. V. J., 401.

Buckley, J. P., 590.

Burke, G. F. 558.

Burns, W. A., 93.

Bullard, J. A., 128.

Buhl, T. D., 255.

Bunting, R. W., 468, 498.

Buttrick, T. R., 240, 241, 246, 349, 545, 583, 587.

Byram, J. Q., 522.

Callahan, J. R., 288.

Campbell, Dr., 368.

Campbell, D. D., 525, 532.

Case C. S., 631.

Cassidy, J. S., 273.

Cassidy, Paul, 222.

Cleland, Jas., 556.

Cook, Geo. W., 87.

Cooke, J. F., 424.

Cotterell, A. J., 209.

Coaglan, T. T., 204.

Covalie, Dr., 134.

Crome, J. N., 85.

Crombie, J., 132.

Cross, H. A., 369.

Cudworth, C. H., 173.

Custer, L. E., 302.

Davis, H. E., 157, 207, 620.

Detwiler, B. B., 207.

Dills, W. B., 99.

Douglas, J., 175.

Dowsley, J. F., 510.

Dubran, E., 131.

Ebersole, W. G., 48.

Emelin, M. J., 472.

Englert, G. A., 627.

Evey, J. M.,621.

Field, G. L., 539, 559.

Fletcher, Thomas, 257.

Foster, M. W., 125.

Garhart, N. K., 285.

Giffen, W. A., 578, 590.

Gillett, H. W., 629.

Gritman, Dr., 620.

Gillett. H. W., 206, 208.

Gilmer, T. L., 122, 163.

Goodale, G. P., 546.

Gowan, W. C., 628.

Graham, Don M., 136,343, 345, 551, 583.

Gray, C. J., 14.

Grove, N. H., 307.

Guilford, S. H., 76.

Hall, L. P., 329, 345.

Hampton, C. G., 568, 588.

Hare, M.D., Geo., 621.

Harlan, A. W., 207, 618.

Haskela, L. P., 630.

Hartzell, T. B., 619.

Herrick, C. A., 161.

Hetrick, 79, 360.

Hey, Mr.. 617.

Hewett, A. C., 631.

Hines, P. F., 34, 36.

Hinman, T. P., 301.

Hoff, N. S., 11, 15, 21, 26, 36, 137, 288, 446, 539.

Hofheinz, R. H., 402.

Hunt, G. E., 123.

Hunter, F. A., 272.

Johnson, C. N., 46, 86.

Keefe, Dr. I. E., 629.

Kennerly, J., 257.

Kimball, C. O., 500.

Kirk, E. C., 39, 124, 372, 399.

Kissel, B. L., 623.

Ladewich, L., 519.

Land, C. H., 29, 34, 175, 184, 186. 244, 247, 343, 528, 584, 587, 595.

Lathrop, H. K., Jr., 554.

LeGro, A. L., 65, 138.

Lemerle, L., 484.

Lemly, Burton, 617.

Lifsey, T A., 625.

Lifsey, Dr., 369.

Linnert, W. F., 417.

Litch, W. F., 404.

Locke, W. S., 276, 282.

Loeffler, E. T., 67, 124, 451, 499.

Logan, F. E., 223, 248.

Long, E. H., 129.

Longfellow, J. C., 308.

Loucks, R. E., 135.

Luke, Dr., 93.

Makum, Dr., 480.

Markham, L. M., 256.

Marshall, M. C., 124.

MaWhinney, Elgin, 619.

McAfee, S. H. 622.

McCardie, W. J., 134.

McDonald, F. W., 341.

McDanald, J. L., 590.

Meyers, P. N., 480.

Miller, Prof. W. D., 377.

Mitchell, G. B., 390.

Moore, E. C., 349.

Moore, Henry I., 618.

Morrow, A. T., 158.

Morse, Dr., 14.

Moyer, W. H. K., 368.

Nelson, J. T., 414.

Newell, V. B., 93.

Nevins, L. W., 130.

Nolan, H. A., 125.

Oakman, C. H., 336, 345.

O'Brian, Dr., 425.

Ottolenguii, R., 401.

Oure, A., 45.

Paden, C. M., 535.

Patterson, J. D., 45, 619.

Patton, C. C., 256.

Perisho, J. P., 257.

Pegram, Jas. W., 128.

Potter, W. H., 127.

Potterf, S. D., 317.

Price, W. A., 300.

Prinz, H., 250, 478.

Prothero, J. H., 20, 22, 31, 35.

Purcell, T. E., 628.

Quimby, C. E., 97.

Quincerat, C. L., 256.

Radcliff, B. T., 622.

Register, N. C., 204.

Reeves, Dr., 625.

Reeves, W. T., 166, 178.

Rogers, G. P., 434.

Roache, F. E., 91, 368, 599.

Ruggles, S. D., 306.

Schuerer, A., 204.

Scheff, D., 135.

Schamberg, M. J., 403.

Semans, H. M., 304.

Shattuck, F., 136.

Shattuck, G. S., 499, 558.

Shannon, E. H., 298.

Sherman, E. C., 299.

Simpson, O. H., 205.

Smith, H. A., 269, 275, 284.

Smith, H. T., 125, 287.

Snow, Dr., 635

Spalding, E. B., 246, 185.

Spooner, Dr., 371.

Stern, D., 311.

Straith, S. S., 112, 139, 349.

Stuart, S. A., 310.

Sweetnam, J. W., 450.

Taggart, W. H., 206, 599.

Talbot, E. S., 37.

Thompson, J. M., 174, 177, 242, 347, 598.

Thompson, J. A., 212.

Thorpe, B. L., 527.

Touchard, F., 139.

Traynham, R. C., 257.

Tracy, W. D., 94.

Travis, J. J., 585, 589.

Turner, C. R., 202.

Tuller, W, N., 315.

Tuller, R. B., 420, 512,

Tweedle, W. H., 157.

Tydings, E. E., 530.

Van Deman, W. H., 296.

Ward, M. L., 66, 136, 235, 240, '245 339, 342, 347, 586.

Watkins, G. B., 247.

Weiss, F. D., 127.

Whithill, R., 506.

Whitslar, W. H., 483.

Wilson, G. H., 426.

Willmott, J. B., 131.

Worboys, C. H., 11, 15, 30, 59, 67, 598.

Wortley, F. G., 197.

Wood, Casey, 10.

Wood, C. P., 348.

Wôodbury, C. F., 408.

Wright, C. M., 270, 384.

Young, D. H., 535.

Zederbaum, Geo., 460, 487.

### ANNOUNCEMENTS.

National Dental Association—Committee on History of Dentistry.—Soon after the Buffalo meeting of the National Dental Association in 1905, this committee announced, through the dental journals and otherwise, the exceptional opportunity which had made it possible to place before the dental profession a comprehensive history of dentistry from the earliest times down to the early days of the nineteenth century. Dr. Vincenzo Guerini of Naples, Italy, well known as a dental historian and archeologist, has placed at the disposition of the committee the results of his labors in the field of dental history, which has formed a large and important part of his life-work.

It was the desire of the distinguished author to bring out this book under the auspices of the National Dental

Association as a material expression of his appreciation of the contributions which America had made to the progress of dentistry as a profession.

This tribute of the author was sympathetically received by the committee on History of the N. D. A., not only because of its exceptional merits and the generous sympathy and appreciation which it expressed, but, further, because it furnished in available form and at once the result for which the committee had been created, and for which its members were individually and collectively laboring.

Upon recommendation of the Committee on History the National Dental Association formally accepted this trust and pledge its moral support to the enterprise of securing as soon as possible the publication of the work of Dr. Guerini.

A thorough canvass of the question from a business point of view disclosed the fact that without a definite, indeed guaranteed market for the book, no publisher could be found willing to undertake the assumed risk of financing the publication, and it was therefore determined by the committee to solicit subscriptions for the work, in order to assure the cost of its publication in advance. Accordingly and based upon careful estimates, the price of subscription was placed at five dollars per copy, and it was found that not less than 700 copies would have to be subscribed for in advance, in order to guarantee the cost of publication.

By the aid of announcements in the journals, by personal solicitation, and direct appeals by circulars, etc., the present total number of subscriptions received by the treasurer is 400, leaving 300 yet to be obtained before the work of publication can be begun.

The committee asks that all who have not yet subscribed will do so at once. Surely there are enough men in our profession who are interested in its history, and willing to devote five dollars to the securing of such an historical record as has never heretofore been attempted. The matter is ready to put in type, and the book can and will be rapidly put through the press just as soon as the amount necessary to accomplish that end is available for use by the committee.

Send your subscription without further delay to Dr. Chas. S. Butler, Treasurer, "The Frontenac," Buffalo, N. Y.

Speak of the matter favorably to your colleagues and induce them to do likewise so that this much-desired object may be consummated without any undue delay. The committee asks that the editors of all dental journals make note of this appeal, and thus lend their important aid to the cause which the committee hopes soon to bring to a successful issue for the benefit of the whole dental profession.

---

Uriah D. Billmeyer, D.D.S., who has for many years practiced dentistry at Chattanooga, Tenn., died at Asheville, N. C., November 26th, from an illness from which he had been suffering for some time.

Dr. Billmeyer was born in 1855 in Michigan. He graduated from the Dental Department of the University of Michigan in 1880, and was for a time an instructor in this college.

He located for practice in Chattanooga, in 1883 where he continued in practice until his illness caused him to go to Ashville for recuperation.

His death was unexpected and came as a great shock to all his friends. He has been an important and useful member of the profession in the South, engaging in general society and professional work, and was for a time a teacher of Operative Dental Surgery in the Dental Faculty of Vanderbilt University, at Nashville.

He will be missed greatly by a host of friends and the profession has lost a faithful and staunch supporter of every thing that is ideal and progressive.

